# Current Status of Newborn Screening in Southeastern Europe

**DOI:** 10.3389/fped.2021.648939

**Published:** 2021-05-07

**Authors:** Vanesa Koracin, Matej Mlinaric, Ivo Baric, Ian Brincat, Maja Djordjevic, Ana Drole Torkar, Ksenija Fumic, Mirjana Kocova, Tatjana Milenkovic, Florentina Moldovanu, Vjosa Mulliqi Kotori, Michaela Iuliana Nanu, Ziga Iztok Remec, Barbka Repic Lampret, Dimitrios Platis, Alexey Savov, Mira Samardzic, Biljana Suzic, Ildiko Szatmari, Alma Toromanovic, Mojca Zerjav Tansek, Tadej Battelino, Urh Groselj

**Affiliations:** ^1^General Hospital Novo mesto, Novo mesto, Slovenia; ^2^University Children's Hospital, University Medical Centre Ljubljana, Ljubljana, Slovenia; ^3^Department of Pediatrics, School of Medicine, University Hospital Center Zagreb and University of Zagreb, Zagreb, Croatia; ^4^Mater Dei Hospital, Msida, Malta; ^5^Department of Metabolism and Clinical Genetics, Institute for Mother and Child Health Care of Serbia, Belgrade, Serbia; ^6^Faculty of Medicine, University of Ljubljana, Ljubljana, Slovenia; ^7^Department of Laboratory Diagnostics, University Hospital Center Zagreb, Zagreb, Croatia; ^8^Department of Endocrinology and Genetics, University Pediatric Clinic, Skopje, Macedonia; ^9^Department of Pediatric Endocrinology, Institute for Mother and Child Health Care of Serbia, Belgrade, Serbia; ^10^Department of Pediatrics, National Institute for Mother and Child Health, Alessandrescu-Rusescu, Bucharest, Romania; ^11^Pediatric Clinic, University Clinical Center, Pristina, Kosovo; ^12^Clinical Institute for Special Laboratory Diagnostics, University Medical Centre Ljubljana, Ljubljana, Slovenia; ^13^Department of Neonatal Screening, Institute of Child Health, Athens, Greece; ^14^National Genetic Laboratory, University Hospital of Obstetrics and Gynecology, Medical University Sofia, Sofia, Bulgaria; ^15^Institute for Sick Children, Clinical Center of Montenegro, Podgorica, Montenegro; ^16^Children Hospital Banja Luka, Banja Luka, Bosnia and Herzegovina; ^17^Children's Clinic, Semmelweis University, Budapest, Hungary; ^18^Department of Pediatrics, University Clinical Center, Tuzla, Bosnia and Herzegovina

**Keywords:** newborn screening, NBS, southeastern Europe, survey, expanded NBS program, neonatal screening, dried blood sample

## Abstract

Significant part of Southeastern Europe (with a population of 76 million) has newborn screening (NBS) programs non-harmonized with developed European countries. Initial survey was conducted in 2013/2014 among 11 countries from the region (Albania, Bulgaria, Bosnia and Herzegovina (BIH), Croatia, Kosovo, Macedonia, Moldova, Montenegro, Romania, Serbia, and Slovenia) to assess the main characteristics of their NBS programs and their future plans. Their cumulative population at that time was ~52,5 million. At that time, none of the countries had an expanded NBS program, while phenylketonuria screening was not introduced in four and congenital hypothyroidism in three of 11 countries. We repeated the survey in 2020 inviting the same 11 countries, adding Cyprus, Greece, Hungary, and Malta (due to their geographical position in the wider region). The aims were to assess the current state, to evaluate the change in the period, and to identify the main obstacles impacting the implementation of expanded NBS and/or reaching a wider population. Responses were collected from 12 countries (BIH—Federation of BIH, BIH—Republic of Srpska, Bulgaria, Croatia, Greece, Hungary, Kosovo, North Macedonia, Malta, Montenegro, Romania, Serbia, Slovenia) with a population of 68.5 million. The results of the survey showed that the regional situation regarding NBS only modestly improved in this period. All of the surveyed countries except Kosovo screened for at least congenital hypothyroidism, while phenylketonuria was not screened in four of 12 countries. Croatia and Slovenia implemented an expanded NBS program using tandem mass spectrometry from the time of last survey. In conclusion, the current status of NBS programs in Southeastern Europe is very variable and is still underdeveloped (or even non-existent) in some of the countries. We suggest establishing an international task-force to assist with implementation and harmonization of basic NBS services where needed.

## Introduction

Newborn screening (NBS) programs include an important set of tests conducted in the early newborn's life aimed at the pre-symptomatic discovery of various rare inborn diseases, where an early detection and treatment is crucial for preventing severe health damage or even death (1). (Available at: https://ec.europa.eu/eurostat/web/population-demography-migration-projections/data). (Available at: https://data.worldbank.org/indicator/NY.GDP.PCAP.CD).

NBS started in the 1960's with Guthrie's test for phenylketonuria (PKU) and gradually expanded to over 50 different diseases in some of the developed countries (2, 3). A tandem mass spectrometry (MS/MS) method has been successfully implemented in the last two decades in many countries allowing fast expansion and simultaneous screening for many diseases from a dried blood spot (DBS) sample (4). New generation sequencing (NGS) is another promising method that can be used for second tier testing and discovery of responsible pathological genetic variants (5). Some of the developed countries are now adding NBS for severe combined immunodeficiency based on T-cell receptor excision circles (TRECs), cystic fibrosis (CF), lysosomal storage disorders (LSD) and others to their NBS (3, 6–8).

Wilson and Jungner described the principles to guide screening decisions, which include available tests, accepted treatments and the cost-effectiveness of the screening, but also emphasize the importance of available facilities for diagnosis and treatment (9). The last-mentioned could be problematic in developing countries, where the lack of financial resources often impedes or even prevents the establishment of screening facilities and employment of appropriately educated staff.

Southeastern Europe (SE Europe) is a heterogeneous region, comprising of developed and developing countries with ~76 million inhabitants. The state of NBS varies significantly between the individual countries. The results of the last study conducted in 2013/2014 showed that four out of 11 countries in the region did not screen for PKU and three of them did not screen for congenital hypothyroidism (CH). At that point, Albania and Kosovo did not have a screening programe. Screening for both PKU and CH existed in Bosnia and Herzgovina (BIH), Bulgaria, Croatia, Moldova, Romania, Serbia and Slovenia, while Macedonia and Montenegro screened for CH only. Screening for congenital adrenal hyperplasia (CAH) was introduced in Bulgaria. At that time none of them used MS/MS for NBS and three of the countries reported plans to implement the MS/MS in planned expansions of NBS (10).

In order to assess the current state of NBS in SE Europe a repeated survey was conducted in 2020 (this time including Cyprus, Greece, Hungary and Malta due to their geographical position in the wider region). Our primary aim was to assess the current state and to evaluate the changes in the NBS in this region in years 2014–2020, and to possibly identify the main obstacles impacting the implementation of expanding the NBS and its outreach.

## Methods

Survey was conducted inviting the identified professionals from 15 countries from SE Europe: Albania, BIH, Bulgaria, Croatia, Cyprus, Greece, Hungary, Kosovo, North Macedonia, Malta, Moldova, Montenegro, Romania, Serbia and Slovenia. Among participants were pediatricians, laboratory geneticists and biochemists responsible for their national NBS programs.

A questionnaire (in Supplementary Material) was designed to assess the main characteristics of NBS of each country, the changes in programs made between 2014 and 2020 and their plans for the future. It consisted of altogether 20 questions, 11 of them asked about the current state of the NBS in the country and eight of them about the possible expansion in the last seven years and in the future. The last question was to provide existing references about the NBS program in each country. The demographics data was obtained using Eurostat website (1) and GDP per capita (in USD) of each country from the World Bank data (2).

The questionnaires were created with the SurveyMonkey® survey platform (SVMK Inc., San Mateo, CA) and distributed to the participants by e-mail. The responses were collected through the same platform and through e-mail. The distribution and collection of the questionnaires took place in November and December 2020 along with final clarifications and data authorization by e-mail. A single response was obtained from each country, except from BIH, where due to organizational aspects, separate responses were collected from the entity of Federation of BIH and the entity of Republic of Srpska (but no response was obtained for Sarajevo). No responses were received from three countries invited (Albania, Cyprus and Moldova). All the responsible participants of the survey were invited as coauthors of the study and have authorized the data provided on behalf of their countries.

## Results

**Figure 1:**
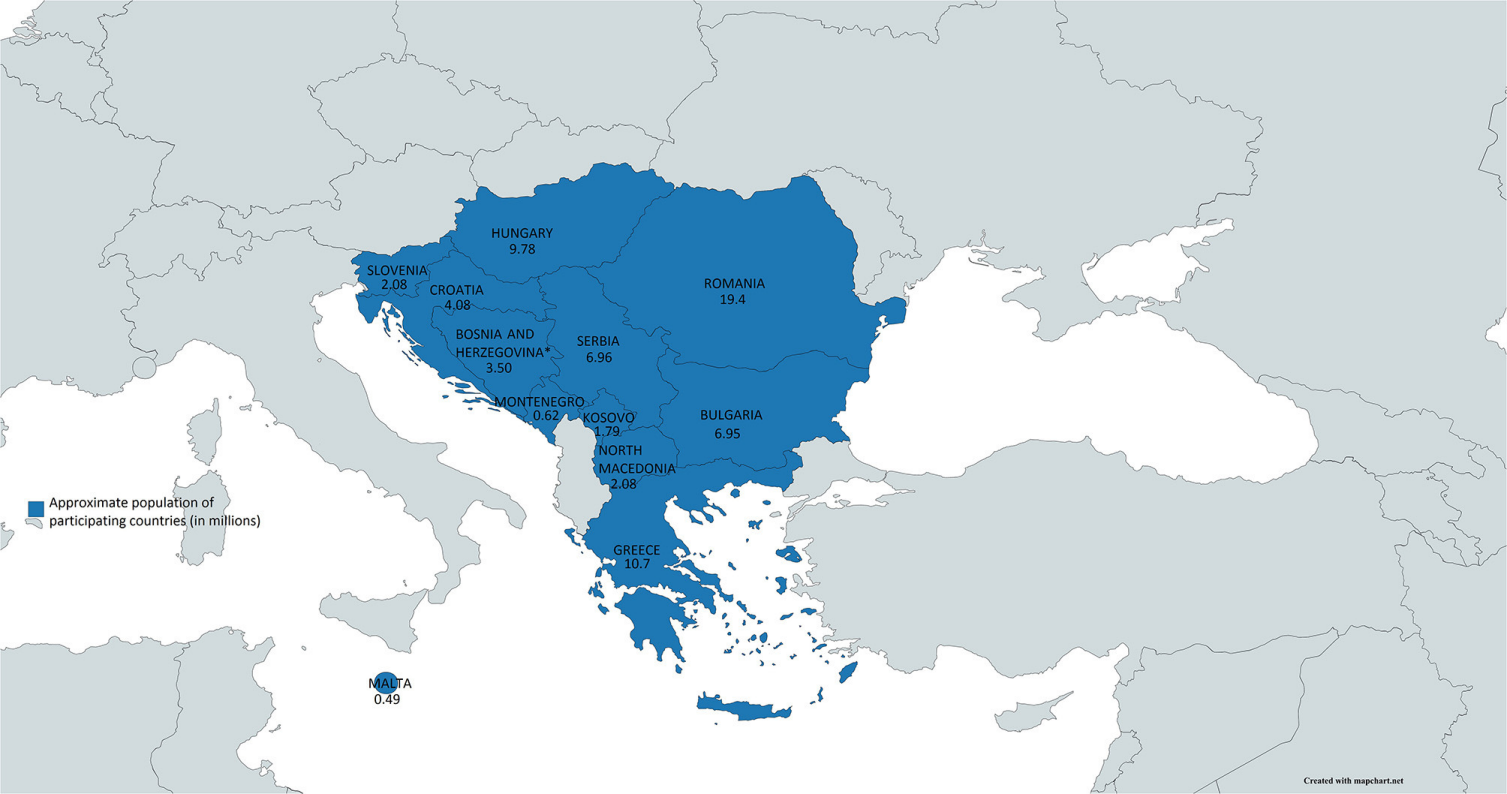
The map of southeastern Europe with countries represented in the survey (in blue) and their populations.

Responses from 12 (out of 15 invited) countries were received. Their cumulative population in 2019 was approximately 68.5 million (Figure 1). The GDP per capita ranged from 29,820 USD (Malta) to 4,420 USD (Kosovo). The number of newborns in 2019 ranged from 188,135 (Romania) to 4,376 (Malta). The number of screening centers in the country ranged from five in Romania to zero in Kosovo (Table 1).

**Table 1:** Demographics and newborn screening programs characteristics in southeastern Europe.

**Country**	**Total pop. in 2019 (Mil.)**	**GDP per cap. in 2019 (USD)**	**Screened/all Nb in 2019**	**No. of screening centers**	**Diseases mandatory screened (year of introduction)**	**Age when screened (h)**	**Lab. methods in NBSP**	**NBS cost (per Nb)**	**Organization of NBS**	**How is the NBS financed**	**International cooperation program on NBS**
BIH—Federation of BIH (without Sarajevo)	3,503^a^	6,110	13,071/13,680	2	CH (2000, 2005)^b^, PKU (2001, 2005)^c^	48–96 h	D, F	6 EUR	RO	NHIS	Yes
BIH—Republic of Srpska	3,503^a^	6,110	9,274/10,180 (all Nb for IEM + PKU controls)	1	CH (2007), PKU (2007)	48–72 h	D, F	22 EUR	EL	NHIS	No
Bulgaria	7,0	9,830	55,315/61,882	2	CAH, CH, PKU (1978–1979),	24 h	F	5 EUR	CW	MH	No
Croatia	4,08	14,930	36,248/36,296	1	CH (1985), CUD (2017), GAI (2017), IVA (2017), VLCADD (2017), LCHADD (2017), MCADD (2017), PKU (1978)	48–72 h	D, TMS (MS/MS), GT for confirmation^d^	19 EUR	CW	NHIS	No
Greece	10,72	19,580	Approx. 83,000/83,763	1	CH (1979), GALT (2006), PKU (1974), G6PD def. (1977)	72–120 h	Genetic Screening Processor (Perkin Elmer), Home (G6PD)	4–5 EUR	CW	MH	Yes
Hungary	9,78	16,730	90,000/90,000	2	CH (1980), CUD (2007), GALT (1975), PA/MMA (2007), GAI (2007), GAII (2007), IVA (2007), VLCADD (2007), LCHADD (2007), MCADD (2007), MSUD (2007), FAH (2007), 3MCC (2007), PKU (1975), BTD (1980), CTNI (2007)	48–72 h	D, F, TMS (MS/MS)	18 EUR	CW	NHIS	No
Kosovo	1,79	4,420	0/26,263	0	/	/	/	/	/	/	Yes
North Macedonia	2,08	6,020	19,408/19,845	1	CH (2007), CF (2018)^e^	48h	D, TMS (MS/MS)	16 - 26 EUR^f^ hospital	CW^g^	NHIS, MH^h^	Yes
Malta	0,49	29,820	3,394/4,376	1	CH (1989), HBP (1989)	72–120 h	D, HPLC	Nd	CW	MH	No
Montenegro	0,62	8,910	7,220/7,223	1	CH (2007)	48–72 h	D	3 EUR	CW	NHIS, MH	No
Romania	19,41	12,920	157,226/188,135	5	CH (2010), PKU (2010)	24–72 h	F, TMS (MS/MS)	4,5 EUR	CW	MH	Yes
Serbia	6,96	7,410	Nd/64,399	1	CH (1983); PKU (1983)	48–72 h	D, F	4 EUR	CW	/	/
Slovenia	2,08	25,940	Approx. 19,000/19,328	1	CH (1981), PKU (1979), CUD (2018), GAI (2018), GAII (2018), PA/MMA (2018), IVA (2018), VLCADD (2018), MCADD (2018), LCHADD (2018), MSUD (2018), FAH (2018), 3MCC (2018), CPDI (2018), CPDII (2018), 3HMGA (2018), HSD (2018), BKT (2018)	48–72 h	D, F, TMS (MS/MS), NGS	9,24 EUR	CW	MH	Yes

*ARG, arginase deficiency; BIH, Bosnia and Herzegovina; BKT, β-ketothiolase deficiency; BTD, biotinidase deficiency; CAH, congenital adrenal hyperplasia; cap., capita; CF, cystic fibrosis; CH, congenital hypothyroidism; CITI, citrullinemia type 1; CITII, citrullinemia type 2; CPDI, carnitine palmitoyltransferase deficiency type 1; CPDII, carnitine palmitoyltransferase deficiency type 2; CTNI, cardiac troponin I; CUD, carnitine uptake defect; CW, country wide; D, Delfia method; EL, entity level; F, fluorimetric method; FAH, tyrosinemia type 1; GAI, glutaric acidaemia type I; GAII, glutaric acidaemia type II; GALT, classic galactosemia; GDP, gross domestic product; G6PD def., glucose-6-phosphate dehydrogenase deficiency; GT, genetic testing; HBP, haemoglobinopathy; HCY, homocystinuria; 3HMGA, 3-hydroxy-3-methylglutaric aciduria; H-PHE, hyperphenylalaninemia; HPLC, high-performance liquid chromatography; HSD, holocarboxylase synthethase deficiency; IEM, inborn errors of metabolism; IVA, isovaleric acidaemia/2-methylbutyrylglycinuria; Lab., laboratory; LCHADD, long-chain L-3-hydroxyacyl-CoA dehydrogenase deficiency/trifunctional protein deficiency; MAL, malonic acidemia; MCADD, medium-chain acyl-CoA dehydrogenase deficiency; 3MCC, 3-hydroxy-methylglutaric aciduria; MET, hypermethioninemia; MSUD, maple syrup urine disease; MH, Ministry of health; Mil., millions; Nb, newborn; NBS, newborn screening program; Nd, no data; NGS, next generation sequencing; NHIS, national health insurance schemes; NKH, non-ketotic hyperglycinemia; No., number; PA/MMA, propionic/methylmalonic aciduria; PKU, phenylketonuria; pop., population; RO, regional organization; TMS (MS/MS), tandem mass spectrometry; TYR, tyrosinemia; VLCADD, very long-chain acyl-CoA dehydrogenase deficiency.*

a *Total population of Bosnia.*

b *2000 in Tuzla Canton, 2005 in Federation of Bosnia and Herzegovina (except Sarajevo).*

c *2001 in Tuzla Canton, 2005 in Federation of Bosnia and Herzegovina (except Sarajevo).*

d *Genetic testing is done in Zagreb only for MCADD—common mutation 985A → G. Other genetic tests for confirmatory purposes are done in laboratories abroad. Organic acids done on GC/MS are used in evaluation of patients positive for GAI, IVA, CUD, MCADD, LCHADD/TFP deficiency and VLCADD (or their mothers).*

e *Selective screening (of 4,001 out of 19,845 newborns in 2019) for PKU, H-PHE, MSUD, CITI, CITII, MET, HCY, ORNT2 mutation, ARG, TYR-I, TYR-II, TYR-III, 3HMGA, NKH, GAI, IVA, PA/MMA, MAL, IBC, BKT, HSD, 3MCC, S-MGAI, TFP (from 2013).*

f *16 EUR in public hospitals, 26 EUR if performed by a private hospital.*

g *CH screening is organized on a state level (coverage of 98%), IEM is covering larger hospitals and covers ~ 1/3 of all newborns in the country, private hospitals additionally send samples abroad (~1,500 per year).*

h *CH screening is completely covered by the MH, other IEM are covered by the MH for public nurseries, NHIS are involved in private nurseries.*

All of the countries except Kosovo screened for CH. Mandatory screening for PKU was not introduced in Kosovo, North Macedonia, Malta and Montenegro. However, North Macedonia reported selective screening for PKU in six bigger nurseries since 2011. Screening for CF was included in the NBS of North Macedonia, Bulgaria screened for congenital adrenal hyperplasia (CAH), Greece screened for glucose-6-phosphate dehydrogenase (G6PD) deficiency and classic galactosemia (GALT). Malta was the only country screening for haemoglobinopathies (sickle cell disease). Expanded NBS (increasing the screening panel of disorders by the use of MS/MS were implemented in Croatia (in 2017, total of eight screened diseases), Hungary (already in 2007, total of 16 diseases) and Slovenia (in 2018, total of 18 diseases) (Table 2).

**Table 2:** Past developments and future plans in newborn screening programs in southeastern Europe.

**Country**	**Expansion of NBS between 2013 and 2019**	**Diseases included in expanded NBS program (year)**	**Diseases planned but unrealised**	**Main obstacles in expanding NBS 2013–2019**	**Plans for further expansion (year)**	**Diseases for further expansion plan**	**Pilot study before further expansion**	**Main obstacles for further expansion**	**Perceived urgency for expanding NBS (1- lowest urgency, 5—highest urgency)**
BIH—Federation of BIH (without Sarajevo)	No	/	/	FR	No	/	/	FR	3
BIH—Republic of Srpska	No	/	CF, CAH, GALT	FR	Yes	CF, GAI, CAH, GALT	Yes	FR	4
Bulgaria	No	/	CF	FR, S, PW	Yes	CF	Yes	FR, S, O,PW	3
Croatia	Yes	MCADD, VLCADD, LCHADD/TFPD, GAI, IVA, CUD (2017)	/	FR, S, O, L	Yes	PA/MMA, HCY, SMA	Yes^a^	FR, S, O, L, incomplete e-Newborn service	4
Greece	No	/	CF	O, L, PW	Yes (2021)	CF, CAH, BTD, expanding the use of TMS (MS/MS)	No	O, L, PW	5
Hungary	No	/	CF	FR, PW	No	/	Yes (CF)	FR	4
Kosovo	No	/	/	FR, PW, country after the war and in process of development	/	/	/	/	5
North Macedonia	Yes	CF (2018), PKU, H-PHE, MSUD, CITI, CITII, MET, HCY, ORNT2, ARG, TYR-I, II, III, 3HMGA, NKH, GAI, IVA, PA/MMA, MAL, IBC, BKT, HSD, 3MCC, S-MGAI, TFP (2013)	Expansion of screening for IEMs to the whole country.	FR, PW	Yes	To first cover the entire country with a screening for IEMs, CAH after that.	/	FR, PW	4
Malta	No	/	PKU	O, L	Yes	CF	Yes	S, O, L	3
Montenegro	No	/	/	FR, S, O, L, SI, PW	No	/	/	FR, S, O, L, SI, PW	3
Romania	No	/	CAH, GAL, CF	FR, O, PW	Yes (2022)	CAH, MSUD, CF, FAH, ASA, CITI, ARG, HPTI, GAI, IVA, 3MCC, PA/MMA, MCADD, LCHADD, TFP, VLCADD, CUD, SCAD, GALT	Yes (2021)	FR, O, PW	5
Serbia	No	/	/	FR, S, O	Nd	/	/	FR, S, O	4
Slovenia	Yes	CUD, GAI, GAII, PA/MMA, IVA, VLCADD, MCADD, LCHADD, MSUD, FAH, 3MCC, CPDI, CPDII, 3HMGA, HSD, BKT (2018)	/	/	Yes (2021)	SMA, SCID, CF, CAH	No	S	4

*ARG, arginase deficinecy; ASA, argininosuccinic aciduria; BIH, Bosnia and Herzegovina; BKT – β-ketothiolase deficiency; BTD, biotinidase deficiency; CAH, congenital adrenal hyperplasia; cap., capita; CF, cystic fibrosis; CH, congenital hypothyroidism; CITI, citrullinemia; CITII, citrullinemia type 2; CPDI, carnitine palmitoyltransferase deficiency type 1; CPDII, carnitine palmitoyltransferase deficiency type 2; CTNI, cardiac troponin I; CUD, carnitine uptake defect; D, Delfia method; EL, entity level; F, Fluorimetric method; FAH, tyrosinemia type 1; FR, lack of financial resources; GAI, glutaric acidaemia type I; GAII, glutaric acidaemia type II; GALT, classic galactosemia; GDP, gross domestic product; G6PD def., glucose-6-phosphate dehydrogenase deficiency; GT, genetic testing; HBP, haemoglobinopathy; HCY, homocystinuria; 3HMGA, 3-hydroxy-3-methylglutaric aciduria; H-PHE, hyperphenylalaninemia; HPLC, high-performance liquid chromatography; HPTI, hypoxantine-guanine phosphoribosyltransferase deficiency; HSD, holocarboxylase synthethase deficiency; IEM, inborn errors of metabolism; IVA, isovaleric acidaemia/2-methylbutyrylglycinuria; L, later management; LCHADD, long-chain L-3-hydroxyacyl-CoA dehydrogenase deficiency/trifunctional protein deficiency; MAL, malonic acidemia; MCADD, medium-chain acyl-CoA dehydrogenase deficiency; 3MCC, 3-hydroxy-methylglutaric aciduria; MET, hypermethioninemia; MSUD, maple syrup urine disease; NBS, newborn screening program; Nd, no data; NKH, non-ketotic hyperglycinemia; O, organization; PA/MMA, propionic/methylmalonic aciduria; PKU, phenylketonuria; PW, lack of political will; S, lack of staff; SCAD, short chain acyl-CoA dehydrogenase deficiency; SCID, severe combined immunodeficinecy; SI, small incidences; SMA, spinal muscular atrophy; TFP, trifunctional protein deficiency; TMS (MS/MS), tandem mass spectrometry; TYR, tyrosinemia; VLCADD, very long-chain acyl-CoA dehydrogenase deficiency.*

a *Expanded NBS still in pilot phase*.

The age of screened newborns ranged from 24 to 120 h, the majority (six) countries started the screening at the age of 48–72 h. In at least three countries with established NBS (Bulgaria, Malta and Romania) more than 10% of the newborns were reportedly not screened (Table 1).

The Delfia method was used in eight countries and the fluorimetric method in four. High-performance liquid chromatography (HPLC) was used for screening for haemoglobinopathy in Malta. Five countries reported the use of MS/MS as a screening method (Croatia, Hungary, North Macedonia, Romania and Slovenia). Croatia used genetic testing for confirming a common mutation in MCADD (985A → G), Greece used genetic screening processor (Perkin Elmer) and Slovenia used NGS as a follow-up test. The cost of screening per newborn ranged from three EUR in Montenegro to 22 EUR in BIH—Republic of Srpska. Furthermore, the cost of screening in North Macedonia if performed by a private hospital reached 26 EUR (Table 1).

Most of the countries reported country wide organization of NBS, while BIH reported regionally organized NBS programs (organized by its three constitutive entities). NBS programs were financed by the Ministry of health in five countries, by the national health insurance schemes in two and by the combination of both in three of them. Five of the countries participated in an international cooperation program on NBS (Table 1).

Five other countries were planning an expansion of the NBS between 2013 and 2019 but could not accomplish it. The main obstacles in expanding the NBS in that period were lack of financial resources, organization and lack of political will (Table 2).

Seven countries plan the expansion of the NBS in the future, six of them are going to conduct a pilot study before the expansion. The urgency to expand the program ranged from three to five (five being the highest urgency and one the lowest), with a median of four. Lack of financial resources, organization and political will continued to be perceived as the main obstacles for expansion (Table 2).

## Discussion

The study assessed the current status of NBS in SE Europe, focused on the characteristics of NBS in each country. The progress from the time of the previous survey done in 2013/2014 was also evaluated (10). The results showed even greater heterogeneity of the NBS in the region than before, considering that Croatia and Slovenia managed to expand the NBS by the use of MS/MS with high coverage (percentage of newborns included), while the basics – for example PKU screening remained sub-optimally implemented in the region, as some of the countries (Montenegro) still did not have a mandatory screening for it (10–12). On the other hand, mandatory screening for CH was successfully implemented in North Macedonia, Montenegro and Romania, where the national registry for CH (MEDILOG) was established in the same year (13–15). The circumstances in Kosovo were worrying, as the NBS was non-existent. The coverage in the region was still not ideal, as more than 10% of newborns were not screened in Bulgaria and Romania. Similar coverage was reached in Malta, where the GDP is approximately three times higher (Table 1). A phenomenon where screening for PKU and some other inborn errors of metabolism (IEMs) by the use of MS/MS was otherwise available in the country but only to newborns in six large nurseries was observed in North Macedonia (10, 16, 17). The main reason for not achieving the goals of expanding the NBS reported in the 2013/2014 survey was for most of the countries' lack of financial resources.

Historically, the NBS in Europe was initiated with smaller programs for screening for PKU during the 1960s, the screening for CH followed a few years later (18, 19). The majority of the SE European countries introduced screening for PKU and CH between 1970s and 1980s, but in BIH, North Macedonia, Montenegro and Romania, the screening was first introduced in the 2000s (Table 1). The only European countries without screening for PKU (Montenegro) and screening for CH (Moldova) are a part of SE Europe. NBS in some form is now present in every European country, except in Albania, Kosovo and Tajikistan (20).

The introduction of MS/MS allowed simultaneous screening for multiple disorders from one DBS and increased the number of amino acidemias, organic acidemias and fatty acid oxidation disorders in the screening panels in the 1990s and first decade of the 21st century (21–23). The first country of SE Europe to expand the NBS with the use of MS/MS was Hungary in 2007, Greece between 2007 and 2009, followed by Croatia (2017) and Slovenia (2018) (5, 10, 24–26) (Table 1). Approximately 50% of the European countries screen for CF and CAH, which were mostly implemented between 2005 and 2010. In SE Europe, CAH is a part of NBS only in Bulgaria. While several SE European countries reported plans for implementing CF in NBS, it was only available as a part of selective screening in some hospitals in North Macedonia at the time of our study (Table 2). Some regions in Italy and the Netherlands started screening for LSD, but they are not a regular part of other NBS programs in Europe (20). Screening for multiple LSD using MS/MS was considered economically justifiable in Hungary in 2012 due to cumulative frequency of LSD similar to acylcarnitine and amino acid IEMs (27).

Modern technologies, such as NGS, were already implemented for CF screening and as a second-tier test in Norway, while the UK conducted a trial of its use as a part of screening algorithm for CF (20, 28, 29). Croatia reported plans for introducing the method as a second-tier as well and Slovenia used it in the pilot study before expanding the NBS in 2018 (5, 20). A survey conducted in 2017 in Bulgaria on potential use of whole-genome sequencing (WGS) in conjunction with the traditional NBS showed that Bulgarian pediatricians and geneticists believed that selective WGS could strengthen their current NBS programs while non-selective WGS for all newborns was not perceived as feasible at that time (30).

Molecular technologies enabled most recent additions to the NBS in some European countries, such as screening for CF, spinal muscular atrophy (SMA) and severe combined immunodeficiency (SCID) (20). Expanding the screening panel with SMA is planned in Croatia and Slovenia, and additionally with SCID in Slovenia (Table 2). To sum up, the screening panels of some countries of SE Europe are already comparable to developed parts of Europe and most of the countries plan on further expansion (25, 26, 31–35).

Secondly, the reported coverage in most of the European countries between 2010 and 2020 was higher than 90%, while the initial coverage in Kyrgyzstan and Turkmenistan, where the NBS was recently established, was 30% (20). In SE Europe over 10% of the newborns are not screened in Bulgaria, Romania and Malta, while the coverage in other countries is over 90% (Table 1). Most of the countries with 100–20,000 newborns per year have one screening laboratory, the number varies due to politico-geographical and socio-economic reasons. Countries from SE Europe with higher-than-necessary number of screening laboratories by that definition are Bulgaria, BIH, Hungary, Romania and Serbia (Table 1) (20). Some of the smaller European countries send the samples to neighboring countries for analysis (e.g., Liechtenstein is covered by Switzerland), which is also done in some parts of Kosovo, where the samples are sent to Serbia (20).

Finally, the decisions on diseases included in NBS are made independently in each European country, as there are currently no policy recommendations or direct oversight at the European level or within the EU (36). Health care has not been included in topics to be governed or overseen by the European Commission, as the member states of the EU consider it to be their own responsibility (20). Therefore, the circumstances regarding NBS in the wider region remain heterogeneous.

The obstacles in comparable regions of the world that lack total NBS coverage are usually poor economies, insufficient health education, lack of government support, early hospital discharge, and large numbers of out-of-hospital births (37, 38).

Similar to countries in SE Europe, parts of Latin America introduced national NBS in the 1990s and the first decade of the 21st century and are working on expanding NBS with MS/MS. The coverage ranges from as low as 1% in Guatemala to 99% in countries with higher socio-economic standards (e.g., Uruguay) but also in Cuba, where NBS is decentralized through more than 175 laboratories (3). Several countries in the Middle East and North Africa (MENA) region have a coverage of screening for CH higher than 90%, while expanded NBS with the MS/MS is often limited or available as a part of selective screening. Nevertheless, it reaches over 90% of the newborns in Israel, that is already considering including SCID in the screening panel, and 100% in Qatar, where the samples are sent to screening laboratory in Heidelberg, Germany (3, 39, 40). In India the challenges are similar, the unsatisfactory state of NBS in one of the countries with largest screening populations has been reviewed and the authors made suggestions to the government for screening implementation, such as convening a central advisory committee to plan for program development, conditions recommended for immediate introduction in urban hospitals, and screening with MS/MS, once a firm infrastructure is in place (37, 38, 41). A model for developing programs in South Asia is the NBS in the Philippines, with 65% coverage, implementation of expanded NBS with MS/MS and even screening for CF (3).

Government prioritization, full or partial government financing, public education and acceptance, health practitioner cooperation/involvement and government participation in program institutionalization were identified as crucial to success for sustainable NBS programs (37, 38).

Despite the small geographical distance, there is a great inequality in the region concerning the level of development of the NBS programs in each country. While some of them still struggle to establish a sustainable screening for PKU and CH, for example Albania and Kosovo, others reach the level of Western Europe and already make plans for including more diseases in already expanded NBS by the use of MS/MS and for introducing NGS as a second tier test (20). Consequently, this could create an even greater divergence between the countries with higher GDP, member states of the EU, and the post-war countries, countries with lower GDP, lack of educated staff and political conditions that do not prioritize good health policies.

The strength of our study was that it included professionals responsible for the NBS in each country and is therefore presenting the first-hand data and experience. As a limitation, we failed to include all the countries in the region, despite making several attempts to reach all the representatives. In addition, the study provides only a partial insight of the state of NBS programs in each country, since we investigated the analytical part of the screening (e.g., screening panels and diagnostic methods used), and omitted the characteristics of the pre-analytical (e.g., taking and derivation of the sample) and post-analytical aspects (confirmation, follow-up and treatment of patients) that are also essential parts of the NBS when it is considered a public health policy.

The current status of NBS in the region of SE Europe is very variable and is still underdeveloped or even non-existent in some of the countries. Furthermore, the situation has not changed very much in the past seven years. A few countries introduced an expanded NBS, while a greater part of them still screen for the CH and PKU only and one of the surveyed countries still does not have a NBS at all. Very recent surveys confirmed a persisting lack of harmonization of NBS programs among European countries, emphasizing the need for more comprehensive guidelines at the European level (20, 42). The urge to put further effort and support into harmonization of the state of NBS in SE Europe through international cooperation and sharing of practical and theoretical knowledge persists. We suggest possibly establishing an international task-force to assist with implementation and harmonization of basic NBS services everywhere needed. Firstly, a careful assessment of the current situation is needed and has to be included in relevant state-of-the art documents and international initiatives. Following from that, more active support in implementing basic standards should be provided, perhaps starting and/or continuing with initiatives to introduce the newborn screening programs where necessary. In addition, a minimal set of disorders to be screened in any specific region could be defined. These efforts could be even more eagerly supported especially by the relevant professional forums, international organizations but also by industry and charities (11).

## Data Availability Statement

The original contributions presented in the study are included in the article/Supplementary Material, further inquiries can be directed to the corresponding author/s.

## Ethics Statement

All the participants agreed to participate in the survey.

## Author Contributions

UG and VK conceptualized and designed the study. UG, VK, and MM carried out the survey and interpreted the results. UG coordinated and supervised data collection and analysis. VK drafted the initial manuscript. UG helped in writing. UG, MM, VK, and TB critically reviewed the manuscript for important intellectual content. All authors approved the final manuscript as submitted and agree to be accountable for all aspects of the work.

## Conflict of Interest

The authors declare that the research was conducted in the absence of any commercial or financial relationships that could be construed as a potential conflict of interest.
