# Supplementary material for: Current Status of Newborn Screening in Southeastern Europe
Source: Front Pediatr. 2021 May 7;9:648939. doi: 10.3389/fped.2021.648939 (PMC8138576; doi:10.3389/fped.2021.648939)
Supplement: Supplementary file 1 [file Data_Sheet_1.PDF]

# CURRENT STATUS OF NEWBORN SCREENING PROGRAMMES IN SOUTHEASTERN EUROPE – 2020 QUESTIONNAIRE

Dear colleague,

We kindly invite you to participate in a survey conducted by the Department of Endocrinology, Diabetes and Metabolic Diseases, University Children's Hospital Ljubljana, to **explore the current state of the newborn screening (NBS) programs in the region of Southeastern Europe**.
The study's purpose is to assess the current state of NBS programs in the region and compare it to the data obtained from the last survey in 2014.

We kindly ask you to complete the brief questionnaire. It should take you approximately 20 minutes. The questionnaire contains questions about the current state of the NBS program in your country, diseases included in the program, plans for the future, and the main obstacles for implementing the expanded NBS program if it is not already implemented.

The results from the last study in 2014 were published in the highly cited article in Molecular Genetics and Metabolism. We plan to **publish this study's results in a theme-focused issue of Frontiers of Pediatrics – section Neonatology, research topic Newborn Screening for Inborn Errors of Metabolism. All respondents will be invited to cooperate as co-authors.** Feel free to contact us through e-mail if you have any questions.

If there is a person in your country who has more direct responsibilities or knowledge in this area, please forward this invitation to him or her if you feel it would be more correct. Ideally we would like to get one merged response from each country, completed by clinician and laboratory geneticist, responsible for the newborn screening program.

**Please complete this questionnaire as soon as possible.** By completing and returning the survey, you give consent for your reply to be incorporated in the study.

Thank you for your participation.

Sincerely,

Urh Grošelj, MD, PhD (urh.groselj.md@gmail.com)
Vanesa Koračin, MD

# CURRENT STATUS OF NEWBORN SCREENING PROGRAMMES IN SOUTHEASTERN EUROPE – 2020 QUESTIONNAIRE

1. Please, fill in **your contact** detail:

| | |
|---|---|
| Name of participant: | |
| Email address: | |
| Position: | |
| Institution: | |
| Country: | |

## CURRENT STATUS OF NEWBORN SCREENING PROGRAMMES IN SOUTHEASTERN EUROPE – 2020 QUESTIONNAIRE

2. What was the **number of all newborns in 2019** in your country?

3. What was the **number of all newborns screened for inborn errors of metabolism in year 2019** in your country?

4. How many **screening centres for inborn errors of metabolism** are in your country?

# CURRENT STATUS OF NEWBORN SCREENING PROGRAMMES IN SOUTHEASTERN EUROPE – 2020 QUESTIONNAIRE

5. What are the **diseases newborns are mandatory screened** for in your country? If implemented, please, fill in the year of first implementation of the screening.

| | |
|---|---|
| CAH – Congenital adrenal hyperplasia | |
| CH – Congenital hypothyroidism | |
| CUD - Carnitine uptake defect | |
| GALT – Classic galactosemia | |
| PA/MMA - Propionic-/methylmalonic acidemia | |
| GAI - Glutaric acidaemia type I | |
| GAII - Glutaric acidaemia type II | |
| IVA Isovaleric acidaemia (IVA)/2-Methylbutyrylglycinuria | |
| VLCADD - Very long-chain acyl-CoA dehydrogenase deficiency | |
| LCHADD - Long-chain L-3-hydroxyacyl-CoA dehydrogenase deficiency/Trifunctional protein | |

| | |
|---|---|
| MCADD - Medium-chain acyl-CoA dehydrogenase deficiency | |
| MSUD - Maple Syrup Urine Disease | |
| FAH - Tyrosinemia I | |
| 3MCC - 3-methylcrotonyl-CoA carboxylase deficiency | |
| PKU – Phenylketonuria | |
| BTD - Biotinidase deficiency | |
| LALD - Lysosomal acid lipase deficiency | |
| CTNL - Citrullinemia / Argininosuccinate synthetase deficiency | |
| SCID - Severe combined immune deficiency | |
| CF – Cystic fibrosis | |
| SMA - Spinal muscular atrophy | |
| Other: | |

6. At what **age** is the **screening for inborn errors of metabolism** performed (in hours)?

7. What are the **laboratory methods** used in **newborn screening program?** (you may check more than one answer to this question)

- ☐ MO Delfia method
- ☐ Fluorimetric method
- ☐ Guthrie's test
- ☐ Sweat test
- ☐ Tandem mass spectrometry (MS/MS)
- ☐ Genetic testing for confirmation
- ☐ Other (please specify)

8. What are the **costs** of **newborn screening program** (per newborn)?

9. On what **level** is the **newborn screening organized?** (you may check more than one answer to this question)

- ☐ Country wide
- ☐ Regionally organized
- ☐ Other (please specify)

10. How is the **newborn screening financed** in your country? (you may check more than one answer to this question)

- [ ] Ministry of Health (MH)
- [ ] National health insurance schemes (NHIS)
- [ ] Combined by both
- [ ] Other (please specify)

11. Do you participate in any **international cooperation program on newborn screening?**

- ( ) Yes
- ( ) No

## CURRENT STATUS OF NEWBORN SCREENING PROGRAMMES IN SOUTHEASTERN EUROPE – 2020 QUESTIONNAIRE

12. Was your **newborn screening program expanded between the years 2013 and 2019**?

- ◯ Yes
- ◯ No
- ◯ Other (please specify)

13. What **diseases were added to the program between the years 2013 and 2019** and when?

14. Are there any **diseases that were planned to be added** to the program but couldn't be realised **between the years 2013 and 2019**?

15. What were **the main obstacles** (please select the most appropriate answer) **in expanding your newborn screening program?**

| | Strongly agree | Agree | Neutral | Disagree | Strongly disagree | N/A |
|---|---|---|---|---|---|---|
| Lack of financial resources | ◯ | ◯ | ◯ | ◯ | ◯ | ◯ |
| Lack of staff | ◯ | ◯ | ◯ | ◯ | ◯ | ◯ |
| Organization | ◯ | ◯ | ◯ | ◯ | ◯ | ◯ |
| Later management | ◯ | ◯ | ◯ | ◯ | ◯ | ◯ |
| Small incidences | ◯ | ◯ | ◯ | ◯ | ◯ | ◯ |
| Lack of (political) will | ◯ | ◯ | ◯ | ◯ | ◯ | ◯ |

Other (please specify)

16. Is there a **further expansion plan for the newborn screening** program in your country? For when is the expansion planned?

- ◯ Yes
- ◯ No
- ◯ Other (please specify)

17. What **diseases are planned to be added to your newborn screening** program?

18. Do you plan to conduct **a pilot study** before implementing an expanded newborn screening or is there one recently going on?

- ◯ Yes
- ◯ No

Please specify:

19. What are **the main obstacles** (please select the most appropriate answer) **to further expand your newborn screening program?**

| | Strongly agree | Agree | Neutral | Disagree | Strongly disagree | N/A |
|---|---|---|---|---|---|---|
| Lack of financial resources | ◯ | ◯ | ◯ | ◯ | ◯ | ◯ |
| Lack of staff | ◯ | ◯ | ◯ | ◯ | ◯ | ◯ |
| Organization | ◯ | ◯ | ◯ | ◯ | ◯ | ◯ |
| Later management | ◯ | ◯ | ◯ | ◯ | ◯ | ◯ |
| Small incidences | ◯ | ◯ | ◯ | ◯ | ◯ | ◯ |
| Lack of (political) will | ◯ | ◯ | ◯ | ◯ | ◯ | ◯ |

Other (please specify)

20. How **urgent is it to expand the newborn screening program** in your country? Please, determine it on the scale (1- the lowest urgency, 5 - the highest urgency).

| 1 | 2 | 3 | 4 | 5 | N/A |
|---|---|---|---|---|---|
| ◯ | ◯ | ◯ | ◯ | ◯ | ◯ |

Other (please specify)

## CURRENT STATUS OF NEWBORN SCREENING PROGRAMMES IN SOUTHEASTERN EUROPE – 2020 QUESTIONNAIRE

21. Please, provide **any references that confirm your answers** to the questionnaire. Please write down the name of the first author, title, journal, year, DOI and Link if possible for every reference.
